# Risk factors and a predictive model for steinstrasse formation after shock wave lithotripsy in pediatric urolithiasis

**DOI:** 10.1007/s00240-026-01975-6

**Published:** 2026-04-10

**Authors:** Ferhat Çoban, Hüseyin Kutlu, Kağan Türker Akbaba, Mehmet Eflatun Deniz, Bedreddin Kalyenci

**Affiliations:** 1 Department of Urology, Faculty of Medicine, Adıyaman University, Adıyaman, Turkey; 2 Department of Biostatistics and Medical Informatics, Faculty of Medicine, Adıyaman University, Adıyaman, Turkey; 3 Department of Urology, İzmir City Hospital, İzmir, Turkey; 4 Department of Urology, Başkent University Adana Dr. Turgut Noyan Training and Research Hospital, Adana, Turkey; 5 Adıyaman University Central Campus Faculty of Medicine Dean’s Office, Altınşehir/Center, Adıyaman, Turkey

**Keywords:** Steinstrasse, ESWL, Pediatric urolithiasis, Machine learning, Explainable artificial intelligence, XGBoost

## Abstract

Steinstrasse is an important complication following extracorporeal shock wave lithotripsy; however, predictive models for risk factors in pediatric populations remain limited. This study aimed to develop and externally validate an explainable machine learning model for predicting steinstrasse formation after ESWL in children. This two-center retrospective study included 807 pediatric patients (< 18 years) who underwent ESWL for renal stones (2015–2025). The internal cohort comprised 685 patients (Adıyaman), while the external validation cohort consisted of 122 patients (Adana). Twenty-six preoperative variables were evaluated. Optimal predictors were identified through consensus-based feature selection using eight algorithms. Thirteen machine learning algorithms were compared using nested cross-validation. Model interpretability was assessed through SHAP, LIME, and partial dependence analyses. The overall steinstrasse incidence was 10.5% (85/807). Nine predictors were selected: S.T.O.N.E. Score, Triple-D Score, age, stone perimeter, stone depth, Hounsfield unit, stone volume, distal ureteral diameter, and skin-to-stone distance. XGBoost demonstrated optimal external validation performance (AUC = 0.949, sensitivity = 90%, specificity = 99.1%, MCC = 0.891) and exhibited excellent generalizability (ΔAUC = 0.001). Explainable artificial intelligence analyses identified age (mean|SHAP|=1.965) and distal ureteral diameter (mean|SHAP|=1.575) as dominant predictors with significant interaction (SHAP = 0.458). Decision curve analysis confirmed clinical utility across a threshold range of 2%–98%. This externally validated model accurately predicts steinstrasse formation using routine preoperative parameters. Integration of patient age, ureteral anatomy, and stone characteristics may guide decisions between ESWL, prophylactic stenting, or alternative endoscopic approaches in pediatric patients.

**Supplementary Information:**

The online version contains supplementary material available at 10.1007/s00240-026-01975-6.

## Introduction

Since its introduction in the early 1980s, extracorporeal shock wave lithotripsy (ESWL) has established itself as an important treatment modality for urinary tract stones due to its minimally invasive nature, high success rates, and favorable complication profile. Currently, the American Urological Association (AUA) and European Association of Urology–European Society for Paediatric Urology (EAU-ESPU) Guidelines recommend ESWL as a first-line treatment option for selected pediatric patients with upper urinary tract stones smaller than 20 mm [1, 2]. However, stone-free rates (SFR) following ESWL vary considerably depending on numerous factors, including stone size, location, chemical composition, and patient-specific anatomical characteristics. SFR reported in the literature ranges from 57% to 92% [3, 4].

With technological advancements, newer-generation ESWL devices have become more compact and comfortable, with treatment sessions being less painful and better tolerated [5]. Nevertheless, ESWL is not entirely free of complications in pediatric patients. Complications reported in the literature include hematuria, pain, and the need for additional procedures, with steinstrasse being one of the clinically notable complications. Less frequently reported complications include urinary retention, fever, asymptomatic and symptomatic hematoma, skin rash, sepsis, and urinoma [6]. Steinstrasse is a condition characterized by the simultaneous alignment of multiple stone fragments along the ureter, occurring in 1.1% to 24.2% of patients in the general population following ESWL. Due to its potential to cause renal colic, significant pain, and infection requiring additional interventions, steinstrasse is considered one of the most important complications associated with ESWL [7, 8].

Although numerous studies have investigated the prediction of steinstrasse development following ESWL in adult patients, data regarding the prediction of this complication in the pediatric population remain limited. The few available studies have reported that steinstrasse risk is associated with factors such as stone size, stone location, and patient age. Current pediatric studies lack a validated and clinically applicable risk assessment tool that comprehensively addresses patient anatomy, geometric and physical stone characteristics, and the interactions among these variables. In this context, our study aimed to comprehensively investigate risk factors associated with steinstrasse development in pediatric patients undergoing ESWL and to develop an explainable machine learning-based prediction model using these factors. The developed model will enable objective and individualized preoperative identification of patients at high risk for steinstrasse development, thereby facilitating patient-specific planning of ESWL, pre-stenting, or alternative treatment approaches to reduce preventable complications. The findings and the developed machine learning model have the potential to support clinical decision-making processes in pediatric patients, enhancing both patient safety and providing a more rational, data-driven approach to ESWL applications.

## Materials and methods

### Study design and population

This retrospective cohort study was conducted to develop and externally validate machine learning models for predicting steinstrasse formation following ESWL, and to determine the importance levels of features affecting steinstrasse formation using explainable artificial intelligence (XAI) methods. The study was conducted in accordance with the Transparent Reporting of a Multivariable Prediction Model for Individual Prognosis or Diagnosis (TRIPOD) guidelines and received approval from the ethics committee of Adıyaman Training and Research Hospital as a two-center study (Decision Number: 2025/6 − 4, Date: June 24, 2025).

Patient data were retrospectively collected from two tertiary referral centers in Turkey where ESWL was performed for renal stones between January 2015 and July 2025. The internal development cohort consisted of 685 patients (84.9%) who met the inclusion criteria among 755 patients screened from Adıyaman Training and Research Hospital, while the external validation cohort comprised 122 patients (15.1%) who met the inclusion criteria among 151 patients screened from Başkent University Adana Dr. Turgut Noyan Hospital Application and Research Center. This geographic diversity was intentionally selected to evaluate the generalizability of the model across different populations and clinical practices.

#### Inclusion criteria

(1) Pediatric patients under 18 years of age; (2) Solitary or multiple renal stones treated with ESWL; (3) Adequate preoperative imaging with non-contrast computed tomography (NCCT) documenting stone location, size, and number; (4) Patients with at least one post-ESWL follow-up visit and documented outcomes.

#### Exclusion criteria

(1) Patients who underwent ESWL for primary ureteral stones; (2) Patients with concurrent ureteral stent (Double-J) placement before or during ESWL; (3) Congenital renal or urinary tract anomalies (horseshoe kidney, pelvic kidney, ureteropelvic junction obstruction, etc.); (4) Cases with secondary stone formation due to metabolic or systemic diseases; (5) Patients with incomplete clinical, imaging, or follow-up data.

The primary endpoint was steinstrasse formation, defined as the accumulation of stone fragments causing ureteral obstruction within 30 days following ESWL.

### Data collection and variables

A comprehensive dataset consisting of 26 preoperative variables was collected from electronic medical records and PACS imaging systems. Non-contrast computed tomography (NCCT) images from both centers were evaluated using the same PACS system, and all measurements were performed using identical measurement tools and standardized methodology. Demographic variables included age, sex, body mass index (BMI), family history of urolithiasis, and personal history of urolithiasis. Stone characteristics were evaluated using NCCT and included: stone location (upper calyx, middle calyx, lower calyx, pelvis), laterality, stone number, and stone dimensions (length, width, and depth in millimeters). Stone perimeter was calculated using the Ramanujan approximation for ellipse perimeter, based on the major and minor axes measured on the axial section where the stone appeared largest, assuming elliptical geometry. In the presence of multiple stones, the perimeter of each stone was calculated separately and summed. Stone surface area was calculated using the ellipse area formula based on the major and minor axes measured on the axial section where the stone appeared largest: π × a × b (where a and b represent half of the major and minor axes, respectively). Stone volume was calculated using the ellipsoid approximation formula (V = 4/3 × π × a × b × c, where a, b, and c represent the semi-axes). Hounsfield unit (HU) values were measured at the center of the stone, and skin-to-stone distance (SSD) was recorded as the shortest perpendicular distance from the stone to the skin surface. Distal ureteral diameter was measured using NCCT images obtained in the pre-ESWL period. Measurements were performed on axial sections at the level immediately proximal to the ureterovesical junction (UVJ), where the distal ureter could be most clearly evaluated. The widest transverse diameter of the ureteral lumen was recorded in millimeters using the inner-to-inner method. Measurements were independently performed by two experienced reviewers, and any discrepancies were resolved by consensus. Sections where lumen boundaries could not be clearly delineated due to transient narrowing from ureteral peristalsis, adjacent vascular structures, or bone artifacts were excluded from measurements.

Clinical scores included S.T.O.N.E. Score (Stone size, Tract length, Obstruction, Number of stones, Essence/density), Triple-D Score (stone Depth × Density × Distance), Quadruple-D Score (Depth × Density × Distance × Diameter), M.A.P. Score, and Guy’s Stone Score (GSS). Hydronephrosis grade (none, mild, moderate, severe) and urine culture status (negative/positive) were also recorded.

### Data preprocessing

#### Missing data and outlier management

Variables with > 20% missing values were excluded from the analysis. Remaining missing values were imputed using k-nearest neighbor imputation (k = 5). Continuous variables were examined for outliers using the interquartile range (IQR) method, and values exceeding 3×IQR beyond the first or third quartile were winsorized to boundary values.

#### Class imbalance management

The steinstrasse endpoint exhibited marked class imbalance with an overall prevalence of 10.5% (internal cohort: 10.9%, *n* = 75/685; external cohort: 8.2%, *n* = 10/122). To address this imbalance while preventing information leakage, resampling was applied only to the internal training data. Six resampling strategies were systematically compared: (1) Original imbalanced data (*n* = 685), (2) Synthetic Minority Over-sampling Technique (SMOTE, *n* = 1,220), (3) Adaptive Synthetic Sampling (ADASYN, *n* = 1,212), (4) Borderline-SMOTE (*n* = 1,220), (5) Edited Nearest Neighbors undersampling (ENN, *n* = 593), (6) SMOTE-ENN hybrid (*n* = 1,037), and (7) SMOTE-Tomek hybrid (*n* = 1,220).

Comparative analysis using 5-fold stratified cross-validation with XGBoost classifier revealed that oversampling methods (SMOTE-ENN: AUC = 0.995; SMOTE-Tomek: AUC = 0.994) achieved the highest internal performance but demonstrated significant overfitting risk. The ENN undersampling approach (*n* = 593) was selected as the optimal strategy due to its superior balance between internal validation performance (AUC = 0.932) and expected generalization capacity, reducing the majority class while preserving minority class distribution (Table 1). Critically, the external validation cohort (*n* = 122) remained completely untouched throughout all preprocessing and training phases.

### Univariate analysis

Prior to machine learning model development, univariate analyses were performed to identify variables significantly associated with steinstrasse formation. Continuous variables were compared between steinstrasse and non-steinstrasse groups using independent samples t-test for normally distributed variables or Mann-Whitney U test for non-normally distributed variables, with normality assessed by the Shapiro-Wilk test. Categorical variables were analyzed using chi-square test or Fisher’s exact test as appropriate. Effect sizes were calculated using Cohen’s d for continuous variables and Cramér’s V for categorical variables, with thresholds of 0.2 (small), 0.5 (medium), and 0.8 (large). Variables with *p* < 0.05 and at least a small effect size were considered statistically significant and clinically meaningful.

### Feature selection

A consensus-based multi-algorithm feature selection strategy was implemented to identify the most robust predictors while minimizing selection bias specific to any single method. Eight complementary feature selection algorithms were applied: (1) Least Absolute Shrinkage and Selection Operator (LASSO) regression with lambda optimization via 10-fold cross-validation, (2) Elastic Net regularization (α = 0.5), (3) Boruta algorithm with Random Forest base classifier (500 iterations, *p* < 0.01), (4) Recursive Feature Elimination (RFE) with XGBoost, (5) Permutation Importance (30 repetitions), (6) Mutual Information-based filter, (7) Model-X Knockoffs with false discovery rate control (FDR < 0.1), and (8) Random Forest mean impurity decrease.

Features selected by at least four of the eight methods (≥ 50% consensus) were retained for model development, ensuring robust and reproducible variable selection. This consensus threshold was determined through sensitivity analysis comparing model performance across different thresholds (3–6 methods).

### Machine learning model development

Thirteen machine learning algorithms spanning diverse methodological families were evaluated: (1) Logistic Regression (baseline), (2) Support Vector Machine (SVM) with radial basis function kernel, (3) k-Nearest Neighbors (KNN), (4) Naive Bayes, (5) Decision Tree, (6) Random Forest, (7) Extra Trees, (8) Gradient Boosting Machine (GBM), (9) Extreme Gradient Boosting (XGBoost), (10) Light Gradient Boosting Machine (LightGBM), (11) Categorical Boosting (CatBoost), (12) Adaptive Boosting (AdaBoost), and (13) Multilayer Perceptron (MLP).

Hyperparameter optimization was performed using nested cross-validation with Bayesian optimization (100 iterations) via the Optuna framework. The outer loop employed 5-fold stratified cross-validation for unbiased performance estimation, while the inner loop used 3-fold stratified cross-validation for hyperparameter tuning. This nested design prevents information leakage and ensures honest estimates of generalization performance. All continuous features were standardized (z-score normalization) within each training fold.

### Model evaluation and external validation

Model performance was assessed using multiple complementary metrics: (1) Area Under the Receiver Operating Characteristic Curve (AUC-ROC) as the primary discriminatory measure, (2) Area Under the Precision-Recall Curve (AUPRC) given class imbalance, (3) Sensitivity (recall) for clinical utility in identifying high-risk patients, (4) Specificity to minimize unnecessary interventions, (5) Precision (positive predictive value), (6) F1-score as the harmonic mean of precision and sensitivity, and (7) Matthews Correlation Coefficient (MCC) for overall balanced assessment.

Overfitting was quantitatively assessed by calculating the AUC difference (AUC_CV – AUC_External), with thresholds defined as: Excellent (difference < 0.05), Good (0.05–0.10), and Overfitting (> 0.10). External validation was performed on the geographically distinct Adana cohort (*n* = 122), which was kept completely separate throughout all model development phases.

### Explainable artificial intelligence (xai) analysis

To enhance model interpretability and clinical applicability, comprehensive explainable artificial intelligence analyses were performed on the best-performing model using the external validation cohort. Six complementary XAI methods were employed:

#### SHapley Additive exPlanations (SHAP)

The TreeSHAP algorithm was used to compute exact Shapley values for each prediction, providing both global feature importance (mean |SHAP|) and local explanations. SHAP beeswarm plots visualized feature value distributions and their directional impact on predictions. SHAP interaction values quantified pairwise feature interactions, identifying synergistic effects.

#### Local Interpretable Model-agnostic Explanations (LIME)

LIME explanations were generated for all external validation samples (*n* = 122) to provide complementary local interpretations. Feature importance consistency between SHAP and LIME was assessed using Spearman rank correlation.

#### Partial Dependence Plots (PDP) and Individual Conditional Expectation (ICE)

PDP curves demonstrated the marginal effect of each feature on steinstrasse probability, while ICE curves revealed individual-level heterogeneity. Two-dimensional PDP interaction plots visualized the joint effects of feature pairs.

#### Accumulated Local Effects (ALE)

ALE plots were computed to address potential correlation-induced bias in PDPs, providing unbiased estimates of feature effects.

#### Permutation Feature Importance

Model dependence was assessed by permuting each feature 30 times and measuring the decrease in AUC, providing a model-agnostic importance ranking with confidence intervals.

#### Global Surrogate Model

An interpretable decision tree was trained to approximate the predictions of the complex model, enabling extraction of simple clinical decision rules. Fidelity (agreement between surrogate and original model predictions) was reported. Anchor-like rule extraction identified minimum sufficient conditions for predictions.

### Statistical analysis

Continuous variables were presented as mean ± standard deviation or median (interquartile range) as appropriate. Categorical variables were reported as frequencies and percentages. 95% confidence intervals for AUC were calculated using the DeLong method. Statistical analyses were performed using Python 3.10 with scikit-learn 1.3, XGBoost 2.0, SHAP 0.42, and LIME 0.2 packages. All tests were two-sided, and statistical significance was set at *p* < 0.05.

## Results

### Study population characteristics

A total of 807 patients met the inclusion criteria and were enrolled in the study. The internal development cohort from Adıyaman Training and Research Hospital consisted of 685 patients (84.9%), while the external validation cohort from Başkent University Adana Dr. Turgut Noyan Hospital Application and Research Center included 122 patients (15.1%). The overall steinstrasse incidence was 10.5% (85/807), with similar rates observed in both cohorts (internal: 10.9% [75/685]; external: 8.2% [10/122]), confirming comparable outcome distributions between centers.

### Univariate analysis results

Univariate analysis identified 17 variables significantly associated with steinstrasse formation (*p* < 0.05). Variables demonstrating at least moderate effect size (Cohen’s d ≥ 0.5 or Cramér’s V ≥ 0.3) included: Triple-D Score (*p* < 0.001, V = 0.346), S.T.O.N.E. Score (*p* < 0.001, V = 0.341), Quadruple-D Score (*p* < 0.001, V = 0.305), age (*p* < 0.001, d = 0.883), skin-to-stone distance (*p* < 0.001, d = 0.787), Hounsfield unit (*p* < 0.001, d = 0.688), stone perimeter (*p* < 0.001, d = 0.565), and stone volume (*p* < 0.001, d = 0.554). Notably, age exhibited the largest effect size among continuous variables, with steinstrasse patients being significantly older (normalized 11.64 ± 4.86 vs. 6.90 ± 5.43, *p* < 0.001). Distal ureteral diameter did not demonstrate univariate significant association (*p* = 0.270, d = − 0.224), suggesting that its predictive value emerges through complex interactions captured by machine learning models (Table 1).

**Table 1:** Univariate Analysis of Variables Associated With Steinstrasse Formation in the Internal Development Cohort (*N* = 685)

Variable	Steinstrasse (−) (*n* = 610)	Steinstrasse (+) (*n* = 75)	*p*	Effect Size	Category
***Continuous Variables***					
Age (years)	6.90 ± 5.43ᵃ	11.64 ± 4.86ᵃ	< 0.001***	d = 0.883	Large
Stone-skin distance (cm)	6.91 ± 2.27	8.74 ± 2.71	< 0.001***	d = 0.787	Medium
Hounsfield unit (HU)	659.0 ± 219.1	809.2 ± 211.8	< 0.001***	d = 0.688	Medium
Stone perimeter (mm)	55.4 ± 13.9	63.2 ± 12.6	< 0.001***	d = 0.565	Medium
Stone volume (mm³)	373.6 ± 275.0	527.0 ± 290.8	< 0.001***	d = 0.554	Medium
Stone surface area (mm²)	61.1 ± 30.4	77.0 ± 30.7	< 0.001***	d = 0.522	Medium
Stone length (mm)	9.96 ± 2.93	11.39 ± 2.83	< 0.001***	d = 0.492	Small
Stone depth (mm)	8.47 ± 2.73	9.81 ± 3.11	< 0.001***	d = 0.482	Small
Stone width (mm)	7.52 ± 2.13	8.49 ± 2.29	< 0.001***	d = 0.455	Small
Distal ureteral diameter (mm)	3.37 ± 1.00	3.15 ± 0.83	0.270	d = − 0.224	Small
BMI (kg/m²)	25.48 ± 3.23	25.53 ± 3.57	0.794	d = 0.014	Negligible
***Categorical Variables***					
Triple-D Score (categorical)	–	–	< 0.001***	V = 0.346	Medium
S.T.O.N.E. Score (categorical)	–	–	< 0.001***	V = 0.341	Medium
Quadruple-D Score (categorical)	–	–	< 0.001***	V = 0.305	Medium
Stone size group	–	–	< 0.001***	V = 0.170	Small
Multiple stones	–	–	< 0.001***	V = 0.146	Small
Guy’s Stone Score	–	–	< 0.001***	V = 0.158	Small
M.A.P. Score	–	–	0.006**	V = 0.154	Small
Urolithiasis history	–	–	0.047*	V = 0.076	Negligible
Gender	–	–	0.091	V = 0.065	Negligible
Hydronephrosis grade	–	–	0.133	V = 0.090	Negligible
Stone location	–	–	0.163	V = 0.087	Negligible
Family history	–	–	0.373	V = 0.034	Negligible
Stone laterality	–	–	0.870	V = 0.006	Negligible
Urine culture	–	–	1.000	V = 0.000	Negligible

Continuous variables are presented as mean ± standard deviation. Group values for categorical variables are presented in supplementary tables (–)

ᵃ Normalized values. *d* = Cohen’s *d* (for continuous variables); *V* = Cramér’s *V* (for categorical variables)

Effect size categories: Large (*d* ≥ 0.8 or *V* ≥ 0.5), Medium (*d* ≥ 0.5 or *V* ≥ 0.3), Small (*d* ≥ 0.2 or *V* ≥ 0.1), Negligible (< 0.2 or < 0.1)

*p < 0.05. **p < 0.01. ***p < 0.001

### Class imbalance management comparison

Systematic comparison of seven resampling strategies revealed significant performance differences (Table 2). Hybrid oversampling methods achieved the highest internal validation metrics (SMOTE-ENN: AUC = 0.995 ± 0.004, F1 = 0.970 ± 0.013; SMOTE-Tomek: AUC = 0.994 ± 0.002, F1 = 0.957 ± 0.008). However, these inflated performance estimates indicated potential overfitting risk. The original imbalanced data demonstrated poor minority class detection (F1 = 0.203 ± 0.081). The ENN undersampling approach (*n* = 593) provided a balanced trade-off (AUC = 0.932 ± 0.025, F1 = 0.623 ± 0.080) and was selected for final model development based on expected generalization capacity.

**Table 2:** Comparison of Class Imbalance Handling Methods

Method	Type	*n*	CV AUC	SD	CV F1	SD
SMOTE-ENN	Hybrid	1,037	0.995	0.004	0.970	0.013
SMOTE-Tomek	Hybrid	1,220	0.994	0.002	0.957	0.008
SMOTE	Oversampling	1,220	0.994	0.002	0.957	0.008
ADASYN	Oversampling	1,212	0.994	0.002	0.959	0.013
Borderline-SMOTE	Oversampling	1,220	0.992	0.003	0.959	0.012
ENN (Selected)	**Undersampling**	**593**	**0.932**	**0.025**	**0.623**	**0.080**
Original (Imbalanced)	–	685	0.892	0.038	0.203	0.081

Performance was evaluated using 5-fold stratified cross-validation with XGBoost classifier on the internal training data (original *n* = 685). The selected method (ENN) was chosen based on expected generalization capability rather than highest internal performance. CV = cross-validation; SD = standard deviation; AUC = area under the receiver operating characteristic curve; SMOTE = Synthetic Minority Over-sampling Technique; ADASYN = Adaptive Synthetic Sampling; ENN = Edited Nearest Neighbors

### Consensus-based feature selection

The multi-algorithm consensus approach identified nine features selected by at least four of the eight methods (≥ 50% consensus). S.T.O.N.E. Score and Triple-D Score achieved the highest consensus (7/8 methods each), followed by age (6/8), stone perimeter (5/8), stone depth (5/8), Hounsfield unit (4/8), stone volume (4/8), distal ureteral diameter (4/8), and skin-to-stone distance (4/8). Notably, the Model-X Knockoffs method with FDR control selected no features; this was an expected outcome given the method’s stringent statistical threshold and the study’s moderate sample size. Nevertheless, the high concordance among the other seven methods supports the reliability of the consensus-based selection. Features excluded from the final model included Quadruple-D Score (3/8), stone surface area (3/8), and demographic variables such as sex, BMI, and family history (≤ 1/8 each) (Table 3).

**Table 3:** Feature Selection Consensus Matrix Across Eight Methods

Feature	LASSO	Elastic Net	Boruta	RFE	Perm.	MI	Knockoffs	RF Imp.	Total
S.T.O.N.E. Score	✓	✓	✓	✓	✓	✓	–	✓	**7**
Triple-D Score	✓	✓	✓	✓	✓	✓	–	✓	**7**
Age	–	✓	✓	✓	✓	✓	–	✓	**6**
Stone perimeter	–	–	✓	✓	✓	✓	–	✓	**5**
Stone depth	–	–	✓	✓	✓	✓	–	✓	**5**
Hounsfield unit	–	–	✓	–	✓	✓	–	✓	**4**
Stone volume	–	–	✓	–	✓	✓	–	✓	**4**
Distal ureteral diameter	–	–	✓	✓	✓	–	–	✓	**4**
Stone-skin distance	–	–	✓	–	✓	✓	–	✓	**4**
Quadruple-D Score	–	–	✓	–	✓	✓	–	–	3
Stone surface area	–	–	✓	–	✓	✓	–	–	3
Stone length	–	–	✓	–	✓	–	–	–	2
Stone size group	–	–	–	✓	–	✓	–	–	2
M.A.P. Score	–	–	–	✓	✓	–	–	–	2
Stone location	–	–	–	✓	✓	–	–	–	2
Gender	–	–	–	–	✓	–	–	–	1
Urolithiasis history	–	–	–	✓	–	–	–	–	1
BMI	–	–	–	–	✓	–	–	–	1
Stone width	–	–	–	–	–	✓	–	–	1
Urine culture	–	–	–	–	✓	–	–	–	1
Multiple stones	–	–	–	–	✓	–	–	–	1
Hydronephrosis	–	–	–	–	✓	–	–	–	1
Guy’s Stone Score	–	–	–	–	✓	–	–	–	1
Stone laterality	–	–	–	–	–	–	–	–	0
Family history	–	–	–	–	–	–	–	–	0

✓ = feature selected by the method; – = feature not selected. Features are ordered by selection count (descending). Features with consensus ≥ 4/8 methods (≥ 50%) were selected for final model development (shown in bold)

LASSO = Least Absolute Shrinkage and Selection Operator; Elastic Net = Elastic Net regularization (α = 0.5); Boruta = Boruta algorithm with Random Forest (500 iterations); RFE = Recursive Feature Elimination with XGBoost; Perm. = Permutation Importance (30 repetitions); MI = Mutual Information; Knockoffs = Model-X Knockoffs with FDR < 0.1; RF Imp. = Random Forest mean decrease in impurity

### Machine learning model performance

#### Internal cross-validation results

Nested 5-fold cross-validation on the internal cohort revealed discriminative performance across gradient boosting ensemble methods (Table 4). SVM achieved the highest internal AUC (0.964 ± 0.022), followed by Logistic Regression (0.963 ± 0.021), CatBoost (0.953 ± 0.014), and AdaBoost (0.951 ± 0.031). XGBoost demonstrated robust performance with the lowest standard deviation among top performers (AUC = 0.950 ± 0.015). Decision Tree exhibited the weakest performance with the highest variance, indicating instability (AUC = 0.804 ± 0.107).

#### External validation results

External validation on the external validation cohort (*n* = 122) demonstrated strong generalization for gradient boosting methods (Table 4). CatBoost achieved the highest external AUC (0.952), followed closely by XGBoost (0.949), LightGBM (0.946), and Gradient Boosting (0.945). Critically, XGBoost demonstrated superior clinical utility with the highest sensitivity (0.90), correctly identifying 9 of 10 steinstrasse cases, while maintaining excellent specificity (0.991) with only 1 false positive among 112 non-steinstrasse patients. The resulting confusion matrix: True Negative = 111, False Positive = 1, True Positive = 9, False Negative = 1.

XGBoost achieved the highest Matthews Correlation Coefficient (MCC = 0.891), indicating excellent overall predictive quality accounting for class imbalance. The precision (positive predictive value) of 0.90 indicates that 90% of patients predicted as high-risk actually developed steinstrasse. Based on this comprehensive evaluation prioritizing clinical sensitivity, XGBoost was selected as the optimal model for deployment and explainability analysis.

#### Overfitting assessment

Quantitative overfitting analysis revealed excellent generalization for most models (Fig. 4). Eleven of thirteen models demonstrated AUC differences below 0.05 (“Excellent” category), including XGBoost (difference = 0.0007), CatBoost (difference = 0.0009), LightGBM (difference = 0.0013), and Gradient Boosting (difference = 0.0061). Two models fell within the “Good” category: Logistic Regression (difference = 0.051) and SVM (difference = 0.056). Notably, Decision Tree showed a negative difference (− 0.036), indicating improved external performance, presumably due to more consistent data distribution in the external cohort. No model exhibited overfitting (difference > 0.10), validating the robustness of the nested cross-validation and ENN resampling approach (Table 4).

**Table 4:** Comparison of Machine Learning Models: Internal Cross-Validation and External Validation Performance

Model	Internal CV		External Validation						Generalization Category
	AUC	SD	AUC	ΔAUC	Sens	Spec	PPV	MCC	
CatBoost	0.953	0.014	0.952	0.001	0.70	0.982	0.78	0.716	Excellent
**XGBoost**	**0.950**	**0.015**	**0.949**	**0.001**	**0.90**	**0.991**	**0.90**	**0.891**	**Excellent**
LightGBM	0.948	0.023	0.946	0.001	0.80	0.982	0.80	0.782	Excellent
Gradient Boosting	0.951	0.015	0.945	0.006	0.70	0.973	0.70	0.673	Excellent
Extra Trees	0.937	0.027	0.937	< 0.001	0.60	0.964	0.60	0.564	Excellent
Random Forest	0.939	0.020	0.930	0.009	0.60	0.964	0.60	0.564	Excellent
MLP	0.951	0.026	0.923	0.028	0.80	0.973	0.73	0.741	Excellent
Logistic Regression	0.963	0.021	0.912	0.051	0.80	0.982	0.80	0.782	Good
AdaBoost	0.951	0.031	0.909	0.042	0.80	0.973	0.73	0.741	Excellent
SVM	0.964	0.022	0.907	0.056	0.70	0.982	0.78	0.716	Good
Naive Bayes	0.895	0.031	0.896	−0.001	0.50	0.848	0.23	0.248	Excellent
KNN	0.879	0.044	0.857	0.022	0.40	0.991	0.80	0.541	Excellent
Decision Tree	0.804	0.107	0.839	−0.036	0.60	0.938	0.46	0.478	Excellent

Internal CV = 5-fold nested stratified cross-validation on internal cohort (*n* = 593 after ENN undersampling); External = independent temporal validation on Adana cohort (*n* = 122). AUC = area under the receiver operating characteristic curve; SD = standard deviation; ΔAUC = Internal AUC − External AUC (positive values indicate potential overfitting); Sens = sensitivity; Spec = specificity; PPV = positive predictive value; MCC = Matthews correlation coefficient. Generalization categories based on |ΔAUC|: Excellent (< 0.05), Good (0.05–0.10). Models sorted by external AUC. XGBoost (bolded) was selected as the optimal model based on highest sensitivity (0.90), excellent specificity (0.991), and superior MCC (0.891) in external validation

#### Model calibration

Calibration of the XGBoost model was assessed using multiple metrics. In internal validation (out-of-fold predictions), the model demonstrated acceptable calibration with a Brier score of 0.046, calibration slope of 0.905, and calibration-in-the-large (CITL) of − 0.019. However, the Hosmer-Lemeshow test yielded a statistically significant result (χ² = 40.53, *p* < 0.001); therefore, Platt scaling was applied for probability recalibration.

**Fig. 1:**
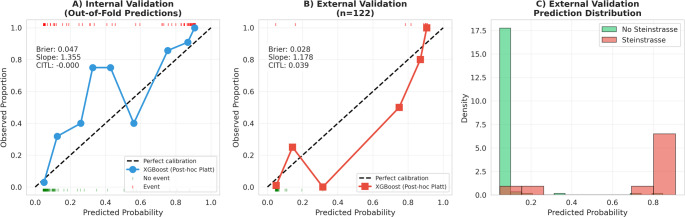
Calibration curves and prediction distribution of the XGBoost model after Platt scaling for Steinstrasse prediction. (**A**) Internal validation calibration curve using out-of-fold predictions (*n* = 593). (**B**) External validation calibration curve (*n* = 122). The dashed diagonal line represents perfect calibration. Rug plots at the top and bottom of panels A and B show the distribution of predicted probabilities for patients with (red) and without (green) Steinstrasse, respectively. Calibration metrics including Brier score, calibration slope, and calibration-in-the-large (CITL) are displayed for each validation cohort. (**C**) Histogram showing the distribution of predicted probabilities in the external validation cohort stratified by actual Steinstrasse outcome, demonstrating good discrimination between risk groups

Following Platt scaling, the model demonstrated good calibration in external validation: Brier score of 0.025, calibration slope of 1.31, and Hosmer-Lemeshow test *p* = 0.727 (Table 5). These results indicate that the calibrated model’s predicted probabilities align well with observed steinstrasse rates. Calibration curves are presented in Fig. 1.

**Table 5:** Comparison of Calibration Methods for XGBoost Model Performance

Calibration Method	Internal Validation			External Validation		
	Brier	Slope	H-L *p*	Brier	Slope	H-L *p*
Uncalibrated	0.045	0.790	< 0.001	0.027	0.705	< 0.001
Platt Scaling	**0.048**	**1.385**	**0.007**	**0.025**	**1.313**	**0.727** ^**ᵃ**^
Isotonic Regression	0.046	1.117	.613^ᵃ^	0.026	1.011	.208^ᵃ^
Post-hoc Platt	0.047	1.355	0.002	0.028	1.178	.691^ᵃ^
Post-hoc Isotonic	0.041	1.009	.508^ᵃ^	0.031	0.663	< 0.001

Brier = Brier score (lower is better, ideal = 0); Slope = calibration slope (ideal = 1.0); H-L *p* = Hosmer-Lemeshow goodness-of-fit test *p*-value (higher indicates better fit). Platt Scaling (bolded) was selected as the optimal calibration method based on the lowest external Brier score and good Hosmer-Lemeshow fit

^ᵃ^ *p* > 0.05, indicating acceptable goodness-of-fit

#### Decision curve analysis

**Fig. 2:**
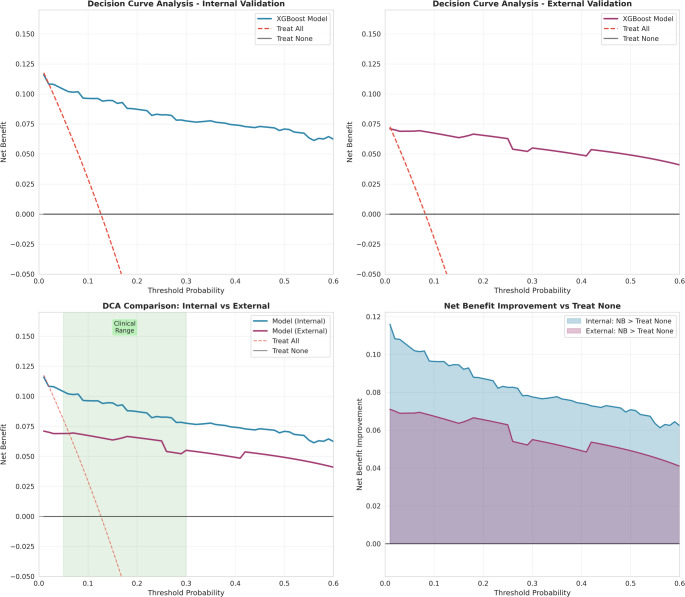
Decision curve analysis evaluating the clinical utility of the XGBoost model for Steinstrasse prediction. (**A**) Decision curve for internal validation cohort (*n* = 593) showing net benefit across threshold probabilities. (**B**) Decision curve for external validation cohort (*n* = 122). The solid blue/purple lines represent the XGBoost model, the dashed red line represents the “treat all” strategy, and the solid gray line at zero represents the “treat none” strategy. (**C**) Comparison of internal and external validation decision curves with the clinically relevant threshold range (5%–30%) highlighted in green. (**D**) Net benefit improvement of the model over the “treat none” strategy, with shaded areas representing the additional clinical value provided by the prediction model. The XGBoost model demonstrated positive net benefit across a wide range of threshold probabilities (3%–98% for internal, 2%–98% for external validation), indicating meaningful clinical utility for risk-stratified decision-making

Decision curve analysis (DCA) was performed to evaluate the clinical utility of the prediction model. The XGBoost model demonstrated positive net benefit across a broad threshold probability range of 2%–98% in both internal and external validation cohorts, indicating robust clinical applicability across diverse clinical scenarios and risk tolerance levels. Within the clinically most relevant threshold range of 5%–30%—corresponding to typical decision thresholds for prophylactic interventions such as pre-ESWL stenting or alternative treatment selection—the model consistently outperformed both “treat all” and “treat none” strategies (Fig. 2). This suggests that using the model for risk stratification would result in better clinical outcomes compared to treating all patients prophylactically or foregoing preventive measures entirely. The sustained net benefit across this threshold range supports the model’s utility for individualized clinical decision-making in pediatric ESWL candidates.

The model provided clinical benefit across threshold probabilities from 3% to 98% in internal validation and from 2% to 98% in external validation. The Net Benefit Integral (NBI) improvement within the clinically relevant threshold range (5%–30%) was calculated as 0.0225 for internal validation and 0.0159 for external validation. These results demonstrate that the XGBoost model provides clinically meaningful predictions capable of supporting risk-based decision-making for steinstrasse prevention in ESWL patient.

### Explainable artificial intelligence analysis

#### Global feature importance

**Fig. 3:**
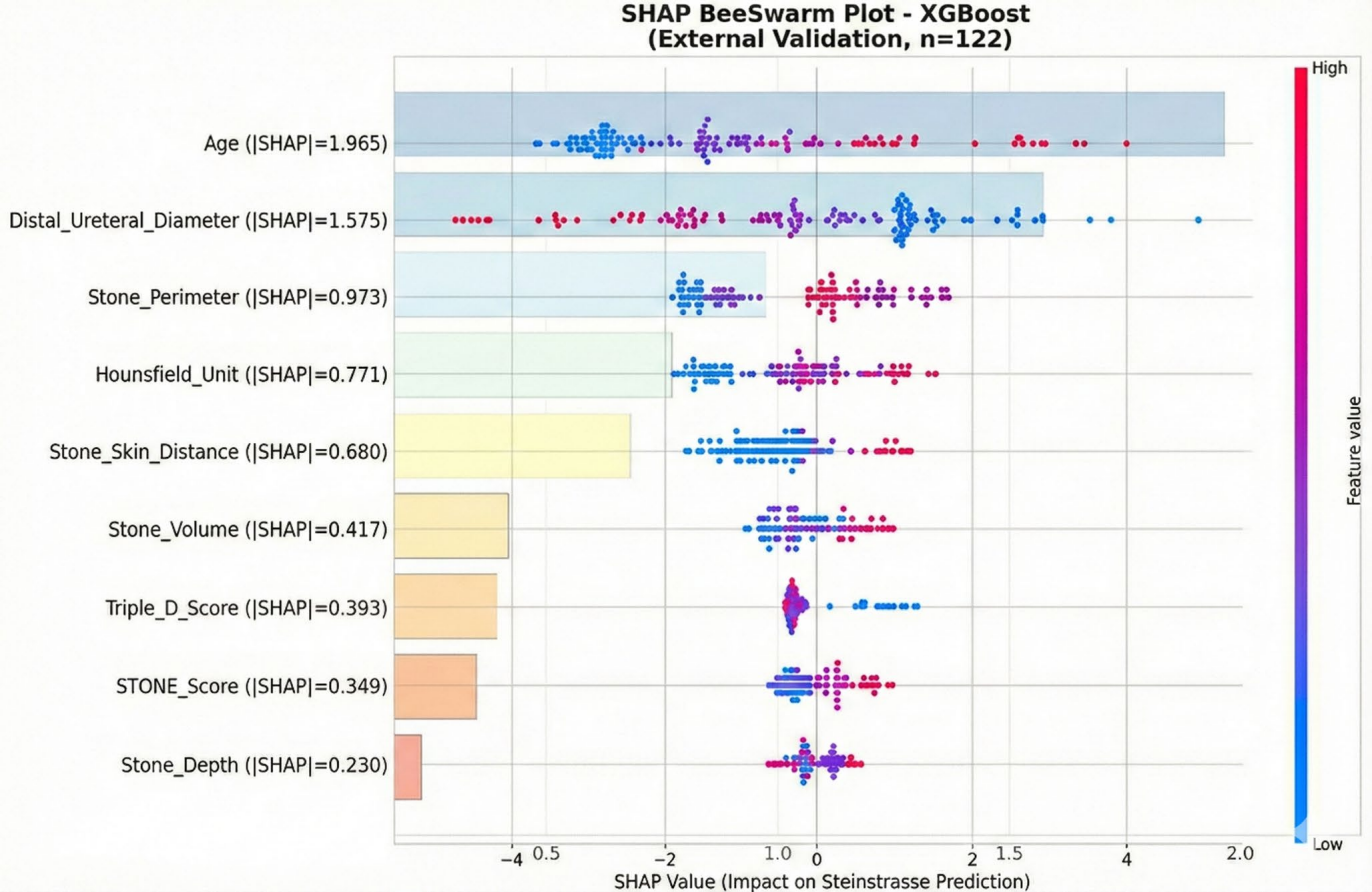
SHAP beeswarm plot showing feature contributions to Steinstrasse prediction (XGBoost, external validation, *n* = 122). Each point represents an individual patient; color indicates feature value (red = high, blue = low), and horizontal position indicates the SHAP contribution to prediction (positive = increased risk, negative = decreased risk)

SHAP analysis on the external validation cohort revealed age as the dominant predictor with mean |SHAP| = 1.965, substantially exceeding other features (Fig. 3). The spread of red points (high age) toward positive SHAP values indicates that advanced age markedly increases steinstrasse risk. Distal ureteral diameter ranked second (mean |SHAP| = 1.575), followed by stone perimeter (0.973), Hounsfield unit (0.771), skin-to-stone distance (0.680), stone volume (0.417), Triple-D Score (0.393), S.T.O.N.E. Score (0.349), and stone depth (0.230).

**Fig. 4:**
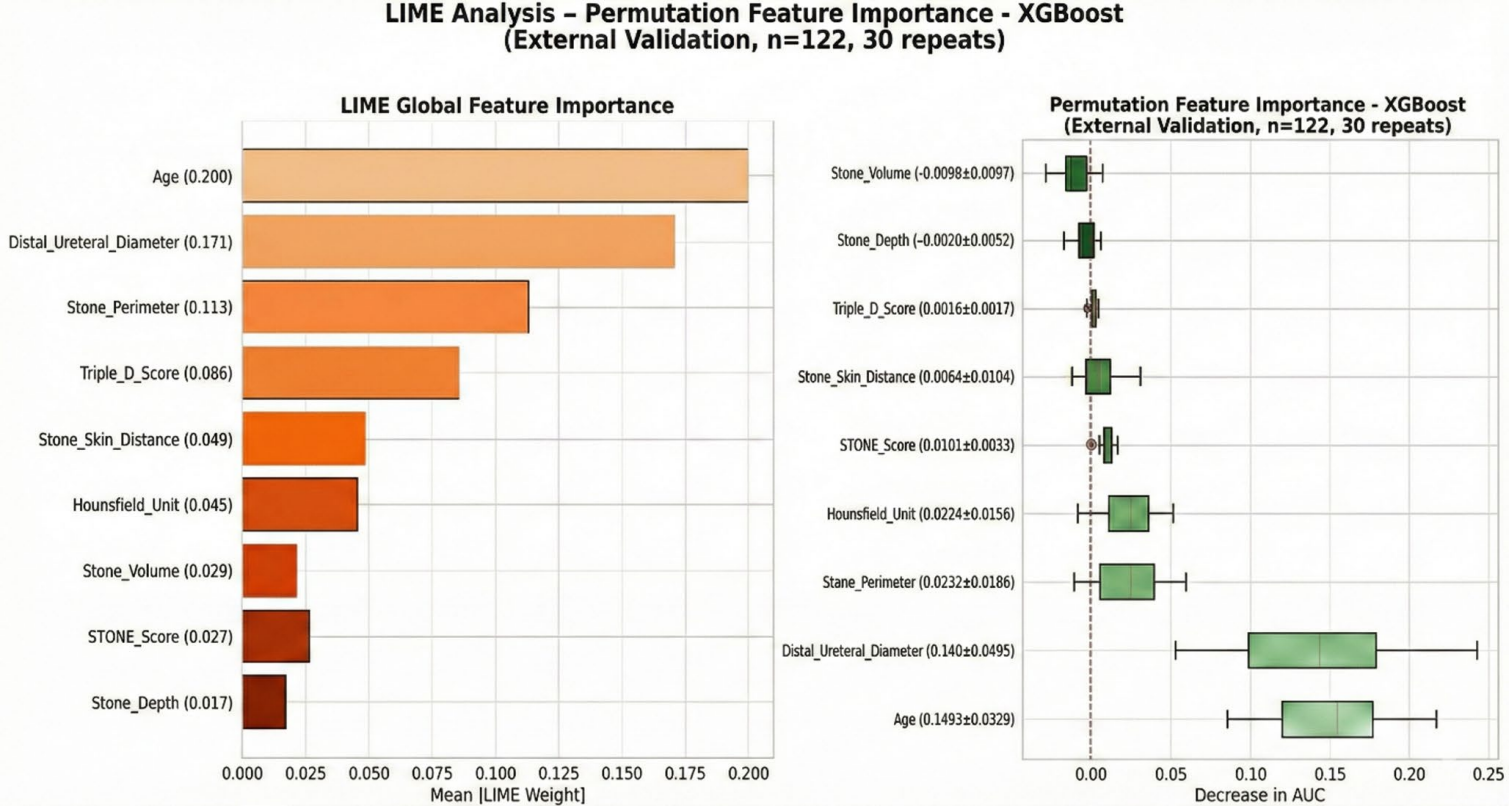
Cross-validation of feature importance using LIME weights (left) and permutation-based AUC decrease (right) for Steinstrasse prediction (XGBoost, external validation, *n* = 122, 30 repetitions)

Permutation importance analysis confirmed the ranking of SHAP values, with age (AUC decrease = 0.149 ± 0.032) and distal ureteral diameter (0.140 ± 0.049) demonstrating the greatest model dependence. LIME analysis independently corroborated these findings, with the top three features (age, distal ureteral diameter, stone perimeter) matching SHAP rankings (Fig. 4). Cross-method correlation analysis demonstrated high concordance: SHAP-Permutation *r* = 0.951, SHAP-LIME *r* = 0.939, Permutation-LIME *r* = 0.915, indicating robust and reproducible feature importance estimates.

Age emerged as a dominant predictor in the model (Fig. 4). To enhance clinical interpretability, the relationship between absolute age (years) and the predicted probability of steinstrasse was visualized (Supplementary Fig. 1). The analysis demonstrated a progressive increase in risk with advancing age, with a noticeable acceleration after approximately 16 years and the highest predicted risk observed in late adolescence.

#### Feature-outcome relationships

**Fig. 5:**
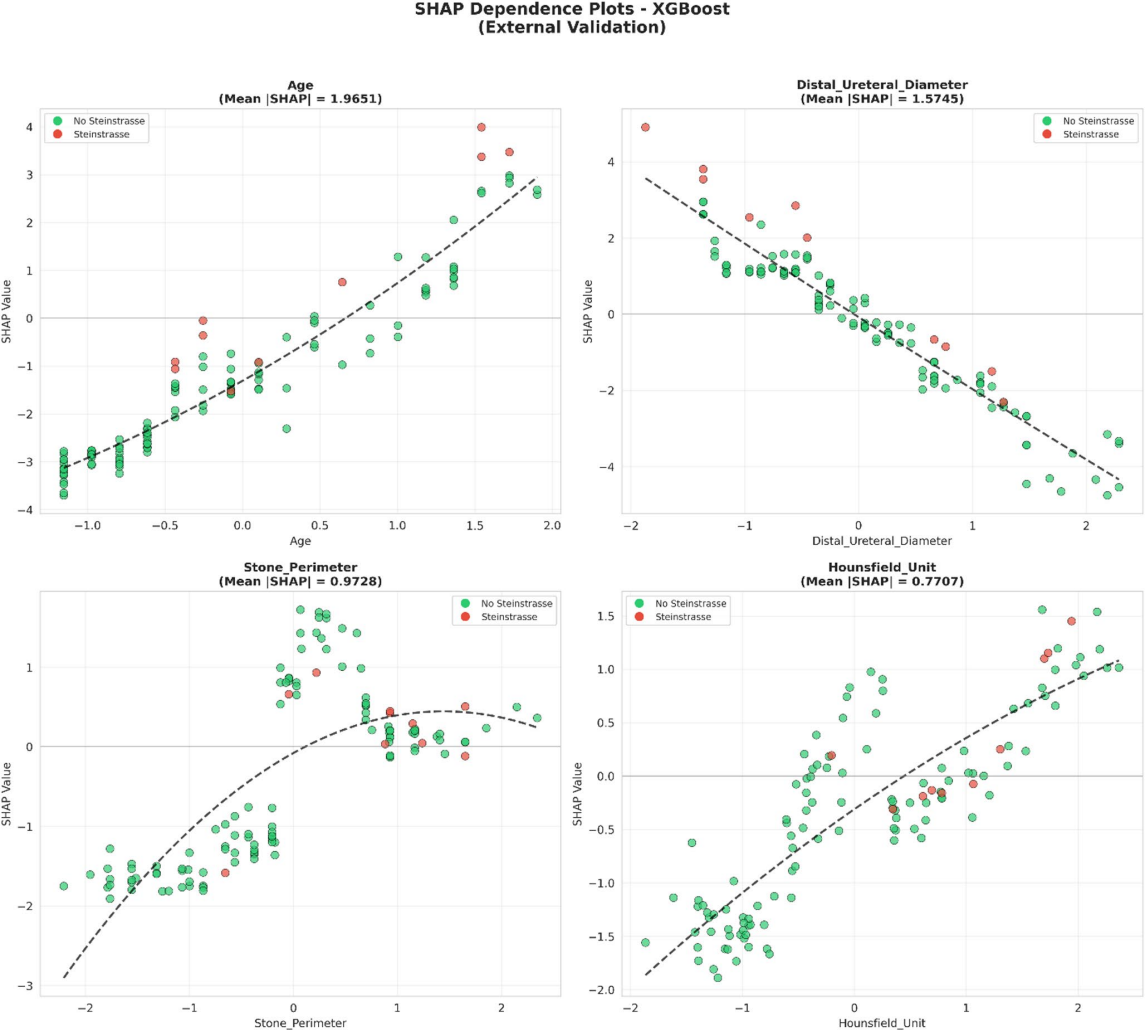
SHAP dependence plots showing the relationship between feature values and SHAP contributions for the top four predictors (age, distal ureteral diameter, stone perimeter, Hounsfield unit) in the external validation cohort. Green and red points indicate patients without and with Steinstrasse, respectively

**Fig. 6:**
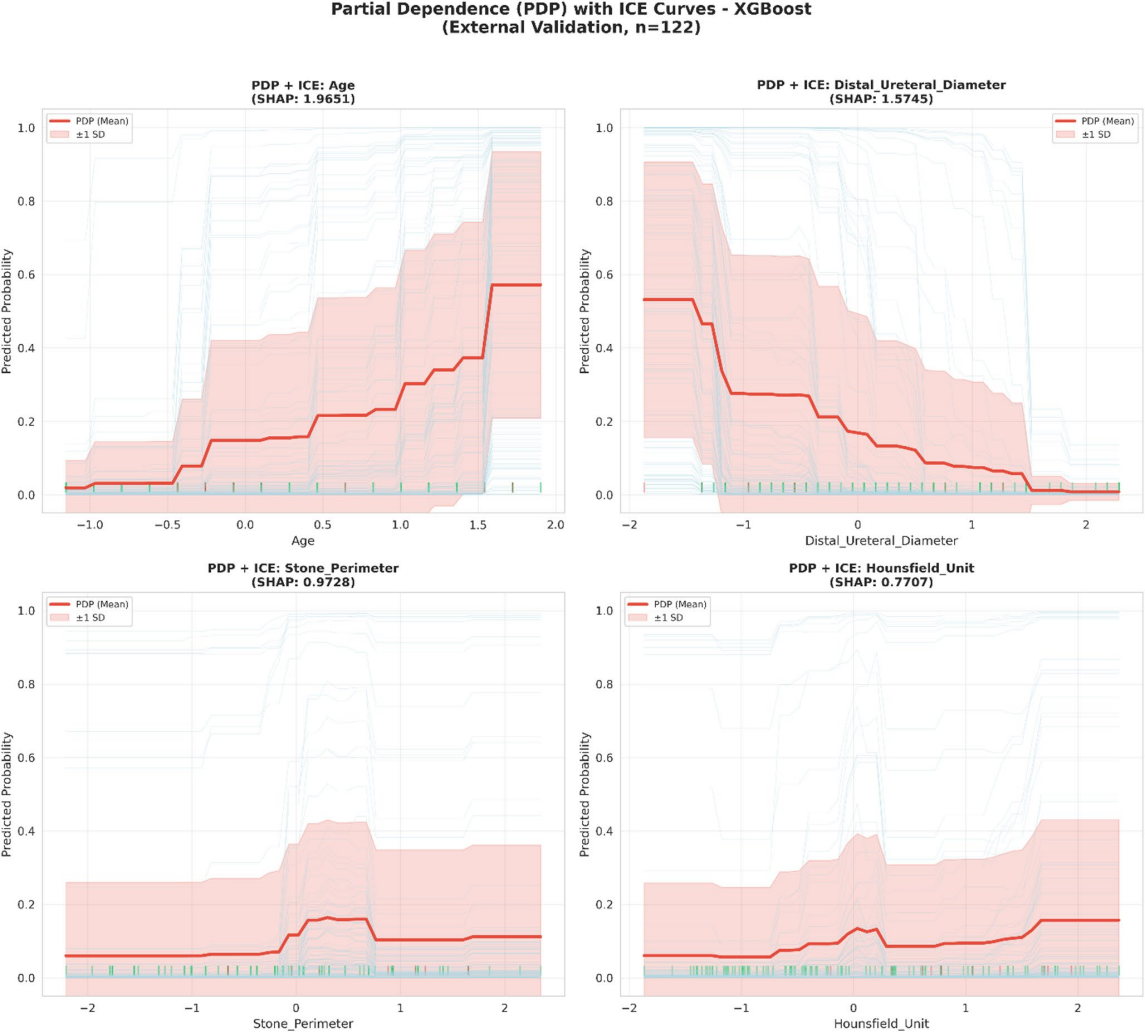
PDP: Partial dependence plots (red line) with ICE: individual conditional expectation curves (light blue) illustrating the marginal effect of top predictors on Steinstrasse probability. Shaded regions represent ± 1 standard deviation

SHAP dependence plots and PDP/ICE curves revealed distinct feature-outcome relationships (Figs. 5 and 6). Age demonstrated a sigmoid-like relationship: steinstrasse probability remained low (< 10%) for younger patients but increased sharply beyond a threshold, reaching > 80% for older patients. Distal ureteral diameter exhibited an inverse relationship, with narrower diameters associated with higher risk, consistent with impaired fragment passage. Stone perimeter and Hounsfield unit displayed positive relationships, with larger and denser stones carrying increased steinstrasse risk.

**Fig. 7:**
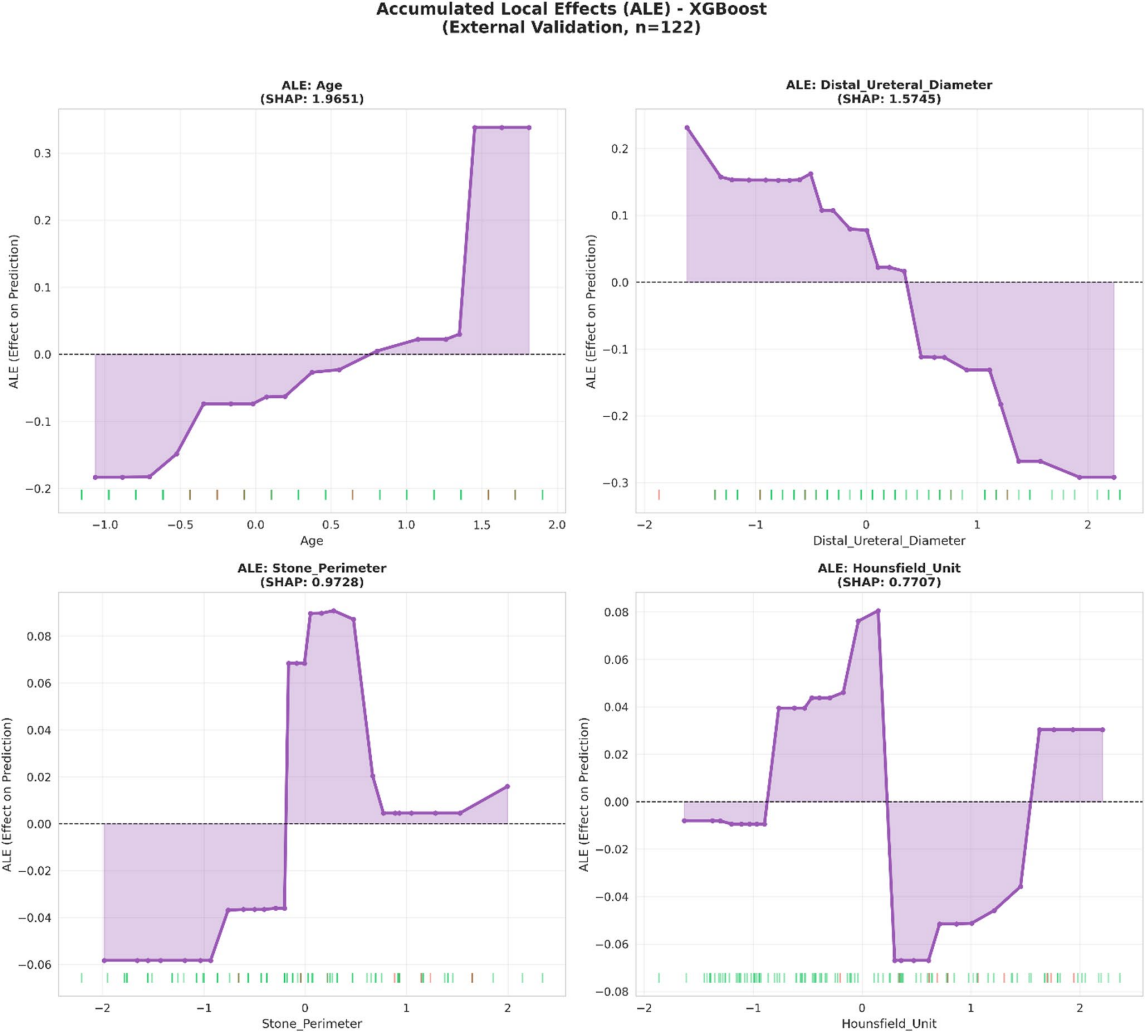
Accumulated Local Effects (ALE) plots illustrating the unbiased marginal effects of top predictors on Steinstrasse prediction after accounting for feature correlations (XGBoost, external validation, *n* = 122)

Accumulated Local Effects (ALE) plots confirmed these relationships while accounting for feature correlations, demonstrating that the age effect ranged from − 0.19 (younger patients) to + 0.34 (older patients) and the distal ureteral diameter effect ranged from + 0.23 (narrowest) to − 0.30 (widest) (Fig. 7).

#### Feature interaction analysis

SHAP interaction analysis revealed clinically meaningful synergistic effects between feature pairs (Fig. 9). The strongest interaction was observed between age and distal ureteral diameter (mean interaction = 0.458), indicating that the effect of age on steinstrasse risk is modulated by ureteral diameter—older patients with narrow ureters face exponentially higher risk. Secondary interactions included age × stone perimeter (0.125), age × stone volume (0.090), and Hounsfield unit × skin-to-stone distance (0.090).

**Fig. 8:**
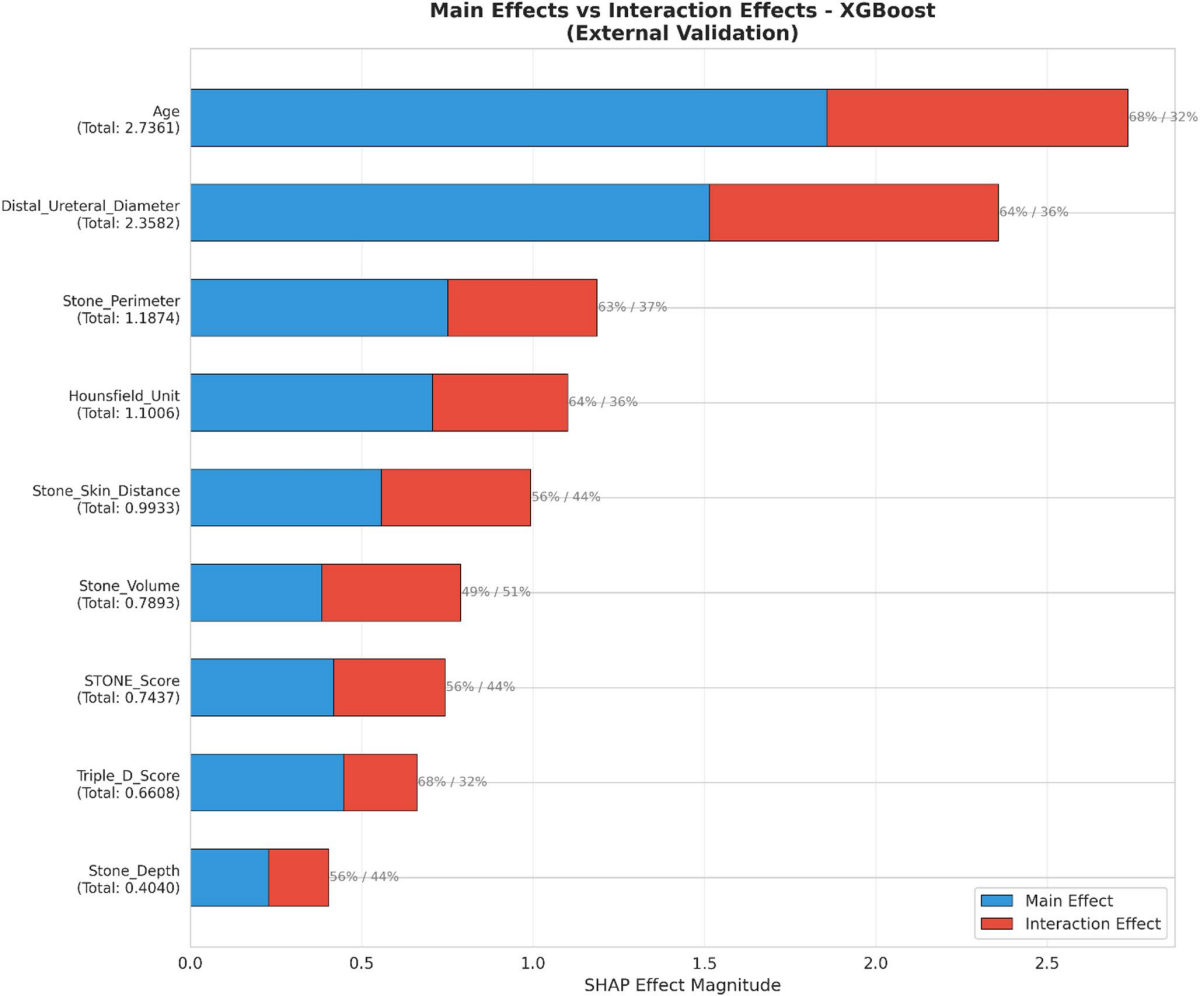
Decomposition of total SHAP effects into main effects and interaction effects for each predictor in the external validation cohort. Percentages indicate the relative contribution of main versus interaction effects to total feature importance

Decomposition of total feature effects into main effects and interaction effects revealed that the contribution of age was predominantly through main effects (68% main, 32% interaction), whereas stone volume exhibited a nearly equal distribution (49% main, 51% interaction), indicating that its predictive value is largely dependent on interactions with other features (Fig. 8).

#### Individual case explanations

**Fig. 9:**
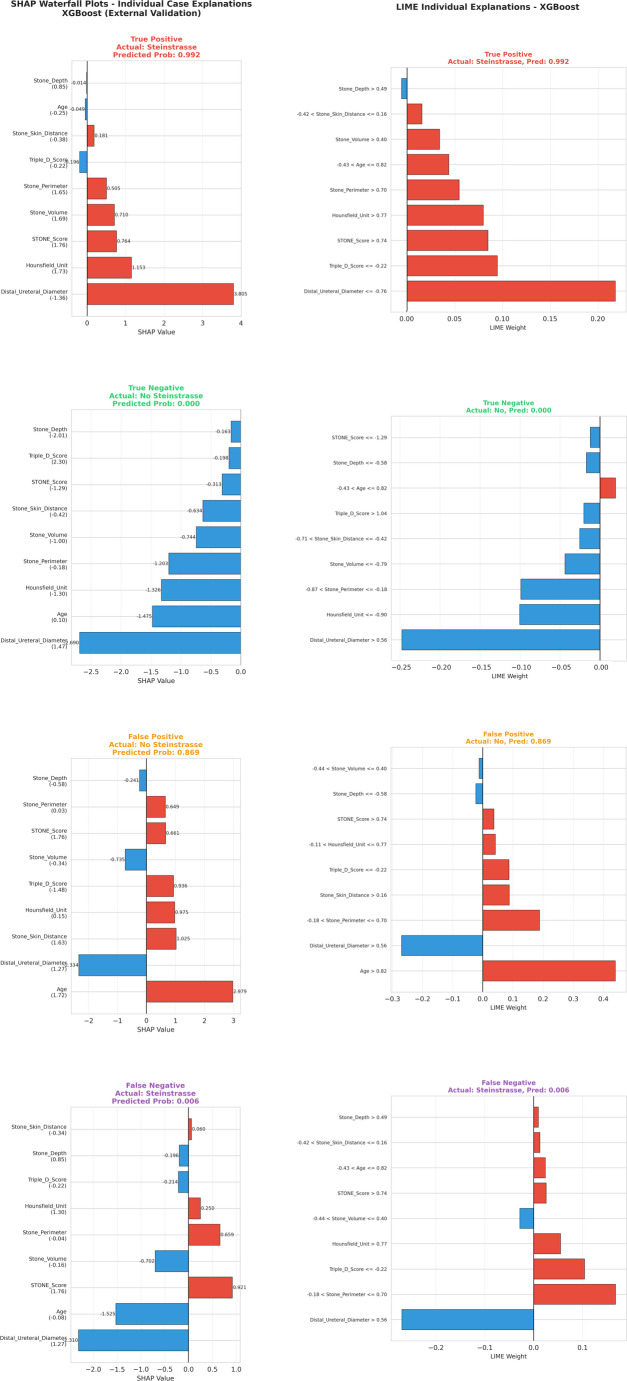
Individual case explanations using SHAP waterfall plots (left) and LIME local interpretations (right) for representative patients across four prediction outcomes: true positive, true negative, false positive, and false negative. Red bars indicate features pushing toward Steinstrasse prediction; blue bars indicate features pushing against it

SHAP waterfall plots provided individual-level explanations for representative cases (Fig. 9). In a correctly identified high-risk patient (True Positive, predicted probability = 0.992), narrow distal ureteral diameter contributed + 3.805 to log-odds, followed by high Hounsfield unit (+ 1.153) and large stone perimeter (+ 0.505). Conversely, in a correctly identified low-risk patient (True Negative, predicted probability = 0.000), wide distal ureteral diameter (− 1.475), young age (− 1.326), and low Hounsfield unit (− 1.203) collectively reduced the predicted risk.

Analysis of the single false negative case (true steinstrasse, predicted probability = 0.006) revealed that wide distal ureteral diameter (− 1.525), young age (− 0.702), and moderate stone perimeter (+ 0.659) produced a misleadingly low-risk profile despite actual steinstrasse occurrence. This case highlights the inherent uncertainty in prediction and underscores the importance of clinical judgment alongside model predictions.

#### Global surrogate model and clinical decision rules

A decision tree surrogate model was trained to approximate XGBoost predictions, achieving 84.4% fidelity (agreement rate) with the original model. The surrogate model used depth = 4 with 13 terminal nodes (leaves), achieving an independent AUC of 0.858 in external validation. Key clinical rules extracted from the surrogate model are as follows:

##### Rule 1 (High Risk)

IF Triple-D Score > − 0.85 (standardized) AND Stone Perimeter > 0.06 AND Age > − 0.52 AND Hounsfield Unit > − 0.69 THEN Steinstrasse = HIGH RISK (50% rate at Leaf 5, *n* = 6).

##### Rule 2 (Moderate Risk)

IF Triple-D Score ≤ − 0.85 AND Distal Ureteral Diameter ≤ 1.47 AND S.T.O.N.E. Score > − 0.78 THEN Steinstrasse = MODERATE RISK (33% rate at Leaf 6, *n* = 9).

##### Rule 3 (Low Risk)

IF Age ≤ 0.37 AND Stone Perimeter ≤ 0.06 THEN Steinstrasse = LOW RISK (0% rate at Leaves 17–23, *n* = 53).

Leaf node analysis revealed three high-risk nodes (Leaf 5: 50% steinstrasse, Leaf 6: 33%, Leaf 24: 27%) and six low-risk nodes with 0% steinstrasse rate. Anchor-like rule analysis confirmed that minimum sufficient conditions for high-risk prediction typically involved at least three features: high Triple-D Score, advanced age, and large stone perimeter or high Hounsfield unit.

### Summary of key findings

In summary, this two-center study demonstrated that machine learning, particularly XGBoost, can accurately predict steinstrasse formation following ESWL with an external validation AUC of 0.949 and sensitivity of 90%. Consensus-based feature selection identified nine preoperative predictors, with age and distal ureteral diameter emerging as the most influential factors through comprehensive XAI analysis. Importantly, the significant interaction between age and distal ureteral diameter (SHAP interaction = 0.458) suggests that older patients with narrow ureters represent a particularly high-risk subgroup requiring intensified surveillance or alternative treatment approaches. The global surrogate model with 84.4% fidelity enables translation of complex predictions into actionable clinical decision rules suitable for bedside application.

## Discussion

This two-center, externally validated study comprehensively evaluated factors predicting steinstrasse formation following ESWL using machine learning and explainable artificial intelligence methods, extending beyond traditional statistical approaches. Our findings demonstrate that steinstrasse risk cannot be explained solely by stone size or classical scores; rather, multidimensional parameters including patient age, distal ureteral diameter, stone geometry (particularly stone perimeter and volume), stone density, and skin-to-stone distance reflecting energy transfer are collectively determinative. Notably, the emergence of age as the most dominant predictor and the strong inverse effect of distal ureteral diameter bring renewed attention to the clinical importance of these variables, which have been addressed in limited or contradictory fashion in the pediatric and adult ESWL literature [9–11].

The role of age in steinstrasse formation following ESWL has been reported with inconsistent results in the literature, particularly in the pediatric population. Previous pediatric studies have reported that younger age is neutral or protective in terms of risk; this has been attributed to the higher elasticity of the ureter in children, more pronounced peristaltic activity, and easier fragment passage [7, 12–15]. However, a cohort analysis studying cystine stone patients—who are considered resistant to ESWL—reported high success rates at younger ages, supporting our discussion of the age-risk relationship [16]. Although data on the independent predictive value of HU in pediatric ESWL are limited, available studies indicate that HU has potential as a parameter that enriches clinical decision-making [17]. Additionally, the fact that stones in younger age groups generally have lower HU values and respond better to ESWL may contribute to the formation of smaller and more regular fragments, thereby reducing the risk of obstruction in the distal ureter. Studies reporting that HU shows particularly lower values in the pediatric patient group and that routine preoperative CT and HU measurement do not significantly contribute to clinical decision-making are also available [18]. However, in most of these studies, age was treated as a linear variable, and parameters that may interact with age, such as ureteral anatomy and stone geometry, were not evaluated in detail. This limited evidence base further justifies our development of multidimensional predictions using machine learning models. In our current study, age emerged as the variable most strongly predicting steinstrasse development, and explainable artificial intelligence analyses demonstrated that the relationship between age and risk is non-linear. Accordingly, steinstrasse probability remained low in younger patients, while risk increased sharply above a certain age threshold, and this increase was particularly pronounced in patients with narrow distal ureteral diameter. These findings suggest that age should be considered not as a standalone risk factor for steinstrasse formation, but rather as an indicator reflecting age-related changes in ureteral biomechanics, stone characteristics, and fragment clearance dynamics. Consequently, the apparently protective effect of young age observed in previous pediatric studies may be attributable to the inadequate demonstration of age-related effects due to analytical methods based on linear assumptions and the neglect of interactions.

The role of distal ureteral diameter in steinstrasse formation following ESWL has not been systematically evaluated in either the pediatric or adult literature. The vast majority of previous studies focused on more easily measurable variables such as stone size, stone burden, and clinical scores; quantitative anatomical measurements of the distal segment of the ureter were generally excluded from analysis [19, 20]. In our study, although distal ureteral diameter did not show a statistically significant association in univariate analysis, it emerged as the second strongest predictor of steinstrasse development in multi-algorithm feature selection and explainable artificial intelligence analyses. This suggests that the effect of distal ureteral diameter manifests not as a linear and isolated relationship, but through complex interactions with other clinical and stone-related variables. Indeed, SHAP, PDP, and ALE analyses demonstrated a distinct inverse relationship between distal ureteral diameter and steinstrasse risk, with this relationship becoming particularly pronounced in older age groups and in conjunction with high stone burden. In other words, when distal ureteral diameter is evaluated in isolation, its effect is masked within heterogeneous patient profiles; however, when considered together with variables such as age, stone geometry, and stone density, it becomes a clinically meaningful determinant. This finding suggests that the failure to identify distal ureteral diameter as a risk factor in previous studies may be largely attributable to statistical methods based on linear assumptions and the neglect of interactions. Our findings reveal that distal ureteral diameter serves as a functional “bottleneck,” significantly affecting fragment clearance following ESWL, and that its inclusion in preoperative risk assessment may provide clinically meaningful contributions, particularly in identifying high-risk patients.

Stone-related factors in steinstrasse formation following ESWL have been evaluated in previous studies mostly through stone size or total stone burden, with limited consideration of the geometric and physical properties of the stone. Although stone size has been reported in the literature as one of the most consistent risk factors for steinstrasse, this parameter has generally been defined through maximum diameter, and geometric measurements reflecting fragment number and alignment have been overlooked. In our study, stone perimeter emerged as a stronger predictor compared to classical stone size measurements, suggesting that stone perimeter better reflects the number of fragments that may form after ESWL and the colonization potential of fragments. Hounsfield unit, representing stone density, has been associated with ESWL success in previous adult studies; however, it has rarely been evaluated in pediatric and steinstrasse-focused studies [3, 9, 21]. Our findings indicate that stones with high Hounsfield units lead to larger and more irregular fragments, thereby increasing steinstrasse risk, and demonstrate that this parameter offers an additional risk layer independent of stone size. Skin-to-stone distance (SSD) has been described in the literature primarily as a factor affecting ESWL success in adults; however, its relationship with steinstrasse development has been examined in limited fashion [22]. The finding in our study that increased SSD is associated with steinstrasse risk suggests that inadequate fragmentation due to reduced energy transfer may predispose to stone street formation in the distal ureter. Finally, although stone size and volume were associated with risk in our study, they showed lower relative impact compared to stone perimeter and Hounsfield unit in explainable artificial intelligence analyses; this indicates that steinstrasse formation should be evaluated not merely by the presence of a “large stone,” but in conjunction with the stone’s geometry, hardness, and effective energy exposure during ESWL. Additionally, one of the most recent pediatric data sources, the retrospective study by Bosnalı et al., demonstrated that pre-ESWL Double-J stenting did not significantly reduce steinstrasse risk [23]. This finding is consistent with previous views suggesting that the fragment passage capacity of the pediatric ureter may differ from that of adults and that stenting may not be necessary in every case. Indeed, the failure to identify variables other than stone size as independent risk factors in multivariable analysis in that study points to the limitations of classical statistical models. Our machine learning-based model, by accounting for interactions of non-stone parameters, demonstrates that stone characteristics together with ureteral anatomy and patient factors collectively constitute risk. The difference between these two approaches emphasizes that steinstrasse risk in the pediatric population should be explained not solely by stone size, but by multidimensional and non-linear interactions.

Various nomogram-based predictive models have been developed in the literature for predicting ESWL outcomes. These studies have generally aimed to predict clinical outcomes using a limited number of variables such as stone size, stone location, Hounsfield unit, and skin-to-stone distance, and have relied on classical regression-based statistical approaches [24]. However, non-linear relationships and interactions among variables have been addressed in limited fashion in these models, and dynamic factors such as ureteral anatomy have mostly been excluded from analysis, particularly in the pediatric population. A systematic review demonstrated that existing nomograms show certain performance in predicting clinical outcomes but have been reported with variable accuracy and limitations across various studies [25]. This finding suggests that the generalizability of classical nomogram-based models in the pediatric population may be limited. Other studies have shown that two different nomograms demonstrate moderate accuracy in predicting stone-free rates; however, it is clear that more effective tools are still needed [26, 27]. In our study, we evaluated the interactions and non-linear relationships of multidimensional variables such as age, distal ureteral diameter, stone geometry, and density using ML + XAI; this approach goes beyond these nomograms based on limited parameters such as stone size, predicting steinstrasse risk more consistently and in an individualized manner.

The main clinical contribution of this study is demonstrating that steinstrasse risk following ESWL can be reliably and individually predicted not by a single parameter, but through the combined evaluation of patient age, distal ureteral diameter, stone geometry, stone density, and skin-to-stone distance reflecting energy transfer. Previous studies have addressed steinstrasse risk mostly through stone size or simple clinical scores, failing to provide a holistic model encompassing the interactions between ureteral anatomy and the physical properties of the stone. Our externally validated machine learning model objectively demonstrates that patients of advanced age with narrow distal ureters, high HU, and stones with large perimeter carry markedly elevated risk for steinstrasse following ESWL. Individualization of pretreatment strategies gains clinical importance in these patients; in high-risk cases, considering prophylactic ureteral stenting or alternative endoscopic treatments such as flexible ureterorenoscopy as the primary option instead of ESWL appears rational. Conversely, in patients with a low-risk profile, the model’s prediction can enhance patient comfort by avoiding unnecessary stenting and invasive procedures. This explainable approach may assist clinicians in identifying patients at higher risk for steinstrasse in the preoperative period and may support individualized risk assessment. However, clinical decisions should be interpreted in conjunction with overall clinical judgment and other patient specific factors[28—30].

The most important strength of this study is presenting a methodologically rigorously designed prediction model with external validation that is clinically interpretable for predicting steinstrasse formation following ESWL. The development of the model using data from two geographically distinct tertiary referral centers and its validation in a completely independent external cohort meaningfully enhances generalizability compared to single-center studies. The application of nested cross-validation to prevent information leakage, addressing class imbalance only on training data, and evaluating model performance with multiple complementary metrics prevented the reported results from being overly optimistic. The consensus approach based on eight different feature selection methods reduced the risk of selected predictors being specific to a single algorithm and strengthened the robustness of the model. Furthermore, the inclusion of anatomical and physical parameters rarely examined in the literature, such as distal ureteral diameter, stone perimeter, and Hounsfield unit, demonstrated that steinstrasse risk cannot be explained solely by stone size or classical clinical scores. Through explainable artificial intelligence analyses (SHAP, LIME, PDP/ICE, and ALE), the decision mechanism of the model was made transparent, and the direction and interactions of predictors were revealed in a manner comprehensible to clinicians.

The retrospective nature of the study may have led to inevitable heterogeneity in data quality and measurement standards. Since distal ureteral diameter and stone-related measurements were based on static imaging data, the dynamic peristaltic properties of the ureter and real-time fragment passage could not be evaluated. Steinstrasse cases were not subcategorized according to clinical severity or intervention requirement, which limited more detailed interpretation of results in terms of clinical spectrum. Additionally, the inclusion of only tertiary centers in the study may create selection bias in terms of patient profile and stone characteristics. Although procedure-specific variables such as energy level, shock wave number, and session number applied during ESWL were available in the dataset, these parameters were deliberately not included in the model development process due to their potential to cause data leakage by being influenced by clinical decisions and early treatment outcomes; this choice aimed to ensure the model can be reliably used in actual preoperative decision-making scenarios. Finally, although external validation was performed, additional validation through larger, multicenter, and prospective studies will further consolidate the clinical applicability and reliability of the model.

## Conclusion

This two-center study demonstrates that steinstrasse formation following ESWL can be predicted with high accuracy using an explainable machine learning model applicable in the preoperative period, incorporating patient age, distal ureteral anatomy, and detailed stone characteristics. This approach, which extends beyond traditional risk assessment based on stone size, may contribute to the reduction of preventable complications by enabling patient-specific and rational decision-making among ESWL, pre-stenting, or alternative endoscopic treatments.

## Supplementary Information

Below is the link to the electronic supplementary material.

Supplementary Material 1

## Data Availability

The data that support the findings of this study are available from the corresponding author upon reasonable request.
