# Supplementary material for: Risk factors and a predictive model for steinstrasse formation after shock wave lithotripsy in pediatric urolithiasis
Source: Urolithiasis. 2026 Apr 10;54(1):86. doi: 10.1007/s00240-026-01975-6 (PMC13068751; doi:10.1007/s00240-026-01975-6)
Supplement: Supplementary file 1 — Supplementary Material 1 [file 240_2026_1975_MOESM1_ESM.docx]

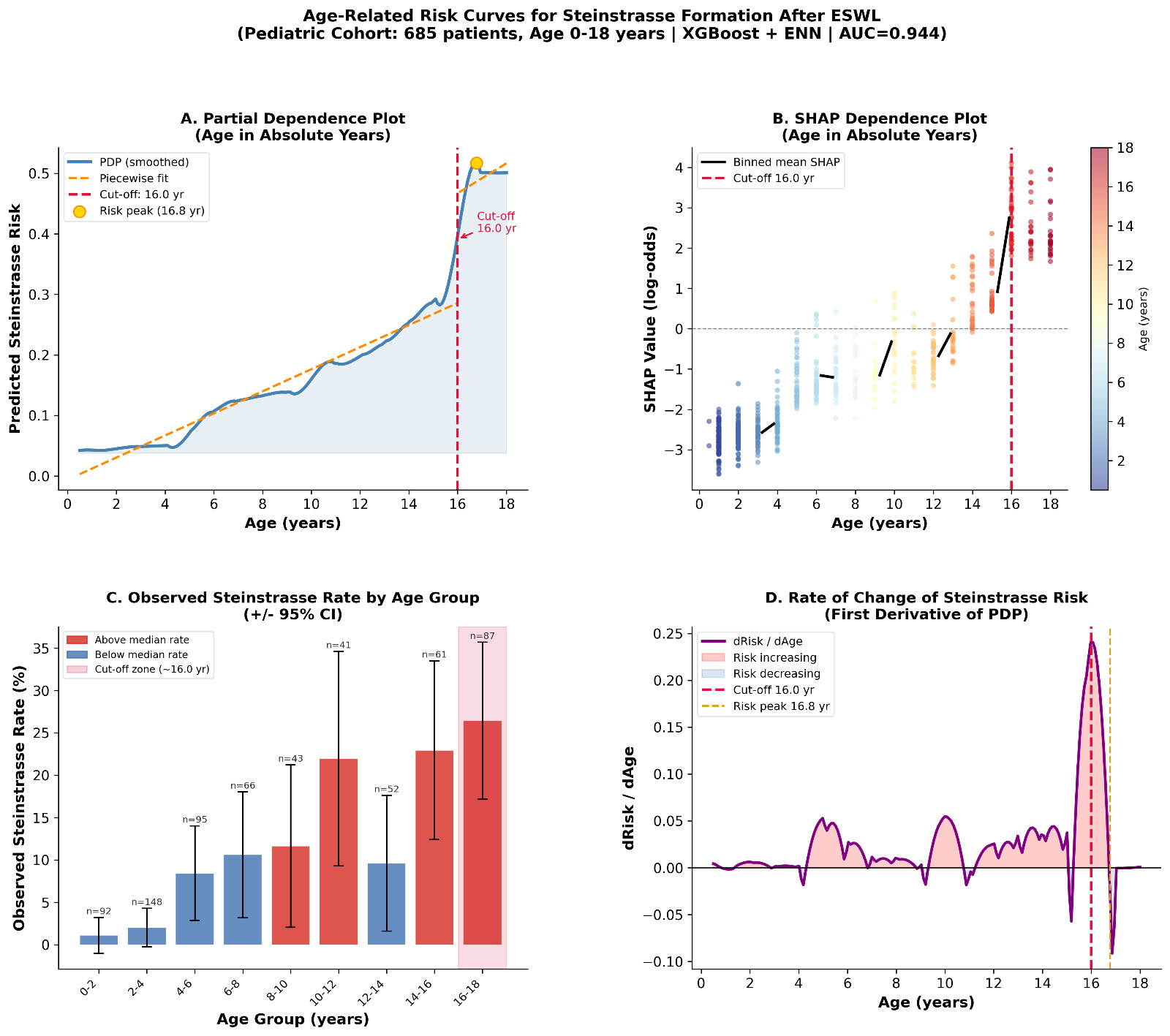
**Supplementary Figure 1.** Relationship between age and the risk of steinstrasse formation after ESWL.

*Panel A (Partial Dependence Plot, PDP): The predicted probability of steinstrasse increases gradually with age up to approximately 16 years (slope ≈ +0.018 per year), followed by a steeper increase thereafter (slope ≈ +0.090 per year). The estimated inflection point occurs around 16 years, with the highest predicted risk observed near 16.8 years.*

*Panel B (SHAP Dependence Plot): SHAP values demonstrate that younger age exerts a protective effect (negative SHAP values). Beginning around 14 years of age, SHAP values approach zero and subsequently become increasingly positive after 16 years, indicating that the model consistently learns age as an important risk factor for steinstrasse formation.*

*Panel C (Observed Event Rates): The distribution of observed steinstrasse rates across age groups shows a similar pattern. The incidence remains relatively low in younger patients (≤16 years; approximately 0.9–22.0%) and increases in older adolescents (>16 years; approximately 26.8%). A gradual upward trend becomes apparent from around 10–12 years of age, with a more pronounced increase after 16 years.*

*Panel D (First Derivative of the PDP Curve): The rate of change in predicted risk remains relatively low and fluctuating between 0 and 15 years, followed by a marked peak around 16 years. This transition supports the presence of a genuine change point rather than a modeling artifact.*
